# Supplementary material for: WordCommentsAnalyzer: A windows software tool for qualitative research
Source: F1000Res. 2018 Sep 4;7:536. Originally published 2018 May 3. [Version 2] doi: 10.12688/f1000research.14819.2 (PMC6137411; doi:10.12688/f1000research.14819.2)
Supplement: Supplementary file 3 [file f1000research-7-17614-s0002.tgz › b2a79496-4ada-47c4-9682-a99ce867836d.zip!/Eight Iranian Health Journals/Journal of Knowledge and Health_30.docx]

© Journal of Knowledge and Health. This work is licensed under CC BY 4.0 (https://creativecommons.org/licenses/by/4.0/).

Volume & Issue: دوره 5، شماره 4: 1389

Title: تأثير تماس پوستي مادر و نوزاد بر اضطراب موقعيتي مادران بستري در بخش سزارين

http://knh.shmu.ac.ir/index.php/site/article/view/153

مقدمه: مطالعه حاضر با هدف بررسي تأثير تماس پوستي مادر و نوزاد بر اضطراب موقعيتي مادران بستري در بخش سزارين بيمارستان شهيد اكبر آبادي تهران انجام شده است. مواد و روش­ها: در يك كارآزمايي باليني تعداد 60 مادر ايراني با سابقه حداقل يك بار عمل سزارين به­طور تصادفي در دو گروه مداخله (اجراي تماس پوستي مادر و نوزاد) و شاهد قرار گرفتند. براي مادران در هر دو گروه، در شيفت صبح، 2 ساعت پس از عمل و پس از ورود به بخش سزارين، طبق روتين، مسكن تجويز مي­شد. در صورتي­كه نمره درد مادران طبق معيار ديداري عددي (VAS) كم­تر يا مساوي 3 (3≤) بود، پرسش­نامه اضطراب اسپيل برگر برای آنان تكميل مي­شد. سپس در گروه مداخله، به مدت 30 دقيقه تماس پوستي اجرا مي­گرديد. در هر دو گروه، 6 ساعت پس از مداخله، در صورتي­كه نمره درد با استفاده از معيار VAS كم­تر يا مساوي 3 بود، مجدداً پرسش­نامه اسپيل برگر تكميل مي­شد. نتایج: شش ساعت پس از اجراي مداخله، تفاوت آماري معناداري در ميانگين نمره اضطراب مادران دو گروه وجود نداشت، اما شدت اضطراب مادران در گروه مداخله به­طور معناداري كم­تر از گروه شاهد بود (037/0=P). شش ساعت پس از اجراي تماس پوستي، میانگین نمره اضطراب مادران در گروه تماس پوستی به­طور معناداری كاهش يافته بود (002/0=P).نتیجه­گیری: نظر به نقش تداوم اضطراب مادر در بروز افسردگي پس از زايمان، و با توجه به نتایج این مطالعه لزوم استفاده از تماس پوستي مادر و نوزاد در بخش­هاي پس از زايمان، به­خصوص بخش سزارين روشن مي­گردد

Title: بررسی مقایسه ای چگونگي مراقبت های دوران بارداری در بارداری های خواسته و ناخواسته

http://knh.shmu.ac.ir/index.php/site/article/view/154

مقدمه: حاملگی ناخواسته یکی از مشکلات کشورهای در حال توسعه مي­باشد. پژوهش حاضر در پاسخ به این سوال که «آیا زنان با حاملگی ناخواسته به­دنبال مراقبت­های پزشکی که نیازمند آن هستند، می­روند؟» انجام گرفته است. مواد و روش­ها: در این مطالعه توصيفي- تحليلي، 270 مادر باردار مراجعه­کننده به بیمارستان­های شهرستان شاهرود جهت ختم بارداری از طریق سرشماری مورد بررسی قرار گرفته و به دو گروه بارداری خواسته و ناخواسته تقسیم شدند. پرسش­نامه مبتنی بر هدف تهیه و از طریق مصاحبه حضوری با مادر و مراجعه به پرونده بهداشتي مادر تکمیل شد. جهت تجزیه و تحلیل داده­ها از آزمون­های کای- دو و من‌ویتنی استفاده شده است. نتايج: نتايج مطالعه نشان داد که بین انجام مشاوره قبل از بارداری، تعداد مراجعات به درمانگاه، زمان مصرف اسید فولیک، مصرف مرتب قرص آهن، انجام آزمایشات روتین بارداری و کسب اضافه وزن مطلوب بین دو گروه بارداری خواسته و ناخواسته تفاوت آماری معناداری وجود دارد (05/0>P). آزمون من‌ویتنی تفاوت معناداری بین ميانگين زمان اولین مراجعه جهت مراقبت­های دوران بارداری (004/0=P) و میانگین تعداد مراجعات به درمانگاه مامایی در حاملگی­های خواسته و ناخواسته را نشان داد (003/0=P). نتیجه‌گیری: از آنجایی که مراقبت­های دوران بارداری از مشکلات حین بارداری و زایمان، پیشگیری می­کند بایستی در رفع موانع آن کوشید. حاملگی ناخواسته یکی از مهم‌ترین عواملی است که نقش اساسی در عدم مراجعه جهت دریافت مراقبت‌های دوران بارداری دارد و بارداری این افراد را پر خطر می­سازد.

Title: بررسی تأثیر اجرای برنامه مدرسه مروج سلامت بر ویژگی¬های سلامت روانی دانش¬آموزان

http://knh.shmu.ac.ir/index.php/site/article/view/156

مقدمه: طرح "مدرسه مروج سلامت" در مدارس کشور به­منظور پیشگیری از سوء مصرف مواد، بروز رفتارهای پرخطر و خشونت­آميز در حال اجرا است. این مطالعه با هدف ارزیابی اثربخشی این طرح در افزایش برخی از شاخص­های سلامت روانی دانش­آموزان شرکت­کننده در طرح فوق انجام گرفت. مواد و روش­ها: در این مطالعه توصيفي- تحليلي تعداد 400 دانش­آموز در مقطع دبيرستان (در قالب 4 گروه (2 گروه مداخله و 2 گروه شاهد) از هر دو جنس دختر و پسر و هر کدام 100 نفر) بر اساس جدول كرجسي و مورگان به شکل نمونه­گیری چند مرحله­ای و تصادفی طبقه­ای انتخاب شدند. ابزار جمع­آوری اطلاعات پرسش­نامه­های استاندارد سلامت عمومي، پرخاشگری، عزت نفس، رضايت از زندگي و شادكامي بود. داده­ها از طریق نرم­افزار آماری SPSS ویرایش 13و از طریق آمار توصیفی و آزمون آماری t زوجي مورد تجزیه و تحلیل قرار گرفت. سطح معناداری 5% در نظر گرفته شد. نتایج: بین میانگین نمرات سطح سلامت عمومی، عزت نفس، شادکامی و رضایت از زندگی گروه آزمایش و گروه مقایسه از نظر آماری تفاوت معناداري وجود نداشت. ولی بین میانگین نمرات عملکرد تحصیلی گروه آزمایش و گروه مقایسه از لحاظ آماری تفاوت معنادار مشاهده شد (005/0P=). نتیجه­گیری: يافته كلي پژوهش حاکی از عدم اثر بخشی آموزش­های ارايه شده در میان دانش­آموزان شرکت­کننده در طرح مدرسه مروج سلامت بر متغیرهای سلامت روان است. بهتر است جهت اثر بخش کردن طرح، شیوه­های اجرایی آن مورد ارزیابی و بازنگری قرار گیرد.

Title: استفاده از گلوتامیک اسید، تریپتوفان و فنیل آلانین به¬منظور تولید نانوذره‌های کلاهک¬دار طلا

http://knh.shmu.ac.ir/index.php/site/article/view/157

مقدمه: مطالعه و بررسی نانوذره‌های طلایی که با استفاده از آمینواسیدها تولید می‌شوند از زمینه‌های بسیار جالب و کاربردی محسوب می‌شوند. آمینواسیدها در این پژوهش هم نقش کاهنده‌ی کاتیون‌های طلا را دارند و هم عاملی برای کلاهک‌دار کردن نانوذره‌های طلا می‌باشند. در واقع اتصال محکم گروه‌های آمینی در آمینواسیدها و پروتئین‌ها به سطوح نانوذره‌های طلا به کاربردهای پزشکی این مواد هیبریدی دلالت دارد. مواد و روش­ها: در این پژوهش نانوذره‌های طلا با استفاده از کاهش محلول حاوی کاتیون‏های طلا با غلظت بهینه 005/0 مولار توسط آمینواسیدهای گلوتامیک اسید، فنیل آلانین و تریپتوفان با غلظت بهینه 025/0 مولار تهیه و عامل‌دار شدند. نتایج: پس از شناسایی این ذره‌ها توسط طیف سنج UV-Vis، غلظت محلول کاتیون‏های طلا و آمینواسیدها بهینه شده و اندازه‌ی ذره‌ها با استفاده از میکروسکوپ الکترونی عبوری و تفرق دینامیکی نور به ترتیب 20-5، 20-10 و 30-20 نانومتر تعیین شدند که مناسب برای فعالیت­های بیولوژکی می‏باشند. نتیجه­گیری: با مقایسه داده‌های حاصل از محاسبات کوانتمی و تجربی مشخص شد آمینواسیدهایی که به‌صورت آنیون به نانوذره‌های طلا متصل می‌شوند از استحکام بیش­تری برخوردارند. نانوذره­های طلای کلاهک دار شده توسط گلوتامیک اسید از آنجایی که دارای گروه‌های کربوکسیلی آزاد هستند، بستر مستعد و مناسبی برای اتصال عوامل زیستی مختلف به این ذره‌ها را فراهم می­کنند.

Title: بررسی وضعیت فلزات سنگین در خاک، آب و گیاه اراضی سبزیکاری شاهرود

http://knh.shmu.ac.ir/index.php/site/article/view/158

مقدمه: ورود فلزات سمی از طریق فعالیت­های انسانی باعث آلودگی بسیاری از خاک­ها شده است. فلزات سنگین از مهم­ترین آلاینده­ها در محیط زیست به­شمار می­روند. این مطالعه به­منظور تعیین غلظت فلزات سنگین در خاک، آب و گیاه اراضی سبزیکاری منطقه شهنما شاهرود انجام شده است. مواد و روش­ها: سبزی­های مورد بررسی شامل سبزی­های برگی جعفری، تره، برگ چغندر، شنبلیله، شاهی، ریحان، تربچه و گشنیز بود. نمونه­برداری طی مدت 3 ماه در فاصله شهریور تا آبان 87 صورت گرفت. تعداد 180 نمونه جمع­آوری شده سبزیجات، آب و خاک، پس از آماده­سازی، توسط دستگاه اسپکتروفتومتر جذب اتمی مطابق با روش استاندارد  مورد آنالیز قرار گرفت. نتایج: مقادیر میانگین غلظت سرب، کادمیوم، کروم، آرسنیک و روی در نمونه سبزیجات به­ترتیب برابر: 99/23، 09/2، 4/5، 26/5، 4/168 میکروگرم بر گرم به ازای بافت گیاه بود. میانگین غلظت فلزات سنگین در آب به­ترتیب: 55/7، 85/4، 78/0، 83/12 و 99/347 میلی­گرم در لیتر نمونه و در خاک به­ترتیب : 12/81، 43/14، 32/ 134، 08/ 19 و 435 میکروگرم به ازاي هر گرم نمونه خاک بود. مقايسه ميانگين غلظت مقادیر فلزات سنگین موجود در سبزیجات، آب و خاک با مقادیر استاندارد اختلاف معناداري را نشان مي­دهد به جز روي كه اختلاف معناداري با مقادير استاندارد نداشت. میزان غلظت سرب، کروم، کادمیوم و آرسنیک در انواع سبزیجات بالاتر از مقدار معمول آن می­باشد. نتیجه­گیری: نتایج تحقیق بیانگر آلودگی آب، خاک و گیاه به فلزات سنگین در اثر مصرف کودهای شیمیایی، دفع غیر بهداشتی فاضلاب و احتراق سوخت­های فسیلی می­باشد.

Title: بررسی اپیدمیولوژیک حوادث در کارگران صنعت ساختمان سازی شهر کرمان

http://knh.shmu.ac.ir/index.php/site/article/view/159

مقدمه: حوادث به­عنوان يکی از مهم­ترين دلايل معلوليت­ها و مرگ در کشورهاي در حال توسعه و توسعه يافته شناخته شده است. در ميان مشاغل و فعاليت­هاي مختلف، صنعت ساختمان­سازي يکي از خطرناک­ترين صنايع در جهان است که میزان خطرات و زیان­های ناشی از آن تا حدی ناشناخته باقی مانده است. هدف از پژوهش حاضر بررسی اپیدمیولوژی حوادث ساختمان­سازی در شهر کرمان می­باشد. مواد و روش­ها: در اين مطالعه توصيفي- تحليلي تمام كارگران ساختماني (153 نفر) كه طي سال­هاي 1385 تا 1387 در شهر كرمان دچار حادثه شغلي شده­اند، مورد بررسي قرار گرفته­اند. اطلاعات اين پژوهش از طريق پرسش­نامه جمع­آوري و داده­ها پس از ورود به نرم­افزار آماري SPSS با استفاده از آزمون­هاي كاي- دو و رگرسيون لجستيك تجزيه و تحليل شده است. نتايج: ميانگين سني افراد حادثه ديده 32 سال (حداقل 16 سال و حداكثر 70 سال) بود كه بيش­ترين ميزان حوادث (55/73%) در گروه سني 16-36 سال رخ داده است. به­طور كلي 3/16 درصد (25 مورد) از حوادث منجر به مرگ شده است و 8/77% از حوادث در افرادي رخ داده است كه داراي تحصيلات راهنمايي و كم­تر بوده­اند. بين سه متغير سال، فصل و شيفت رخ داد حادثه با نتيجه حادثه (مردن/زنده ماندن) ارتباط آماري معنادار وجود دارد اما بين وضعيت بيمه هر فرد با نتيجه حادثه ارتباط معناداري مشاهده نشده است (08/0=P). نتيجه­گيري: در ایران ارتباط قوی میان دلایل و نتایج حوادث وجود دارد، لذا این مطالعه پیشنهاد بررسي بيش­تر با هدف تغییر در روند موجود را جهت پیشگیری از حوادث در آینده ارايه می­نماید.

Title: بررسی وضعیت سرولوژیک و جنبه¬های اپیدمیولوژیک مواجهه شغلی با ویروس¬های هپاتیت B و Cدر پرسنل بهداشتي- درماني

http://knh.shmu.ac.ir/index.php/site/article/view/160

مقدمه: كاركنان سیستم بهداشتي- درماني كه با خون و مايعات بدن در تماس مي­باشند، افراد با خطر بالا براي كسب عفونت­هاي منتقله از راه خون مي­باشند. هدف مطالعه حاضر، بررسي وضعيت سرولوژیک از نظر عفونت­هاي هپاتيت C و  Bو تعيين جنبه­های اپیدمیولوژیک موارد مواجهه شغلی در كاركنان شاغل در بيمارستان فاطميه شاهرود مي­باشد. مواد و روش­ها: در این مطالعه توصیفی، 191 نفر از پرسنل بيمارستان فاطميه شاهرود اعم از کادر درماني و اداري مورد بررسي قرار گرفتند. بعد از دادن توضيحات لازم، cc5 نمونه خون جهت تست­های HBS-Ag، HBS-Ab و HCV-Ab به روش الیزا گرفته شد و هم­زمان سابقه كاري، سابقه واکسیناسیون هپاتیت B، سابقه تماس با خون و مايعات بدن ثبت شد. نتايج: متوسط سن افراد شركت­كننده در مطالعه 9±34 سال بود. بيش­ترين موارد تماس با خون و مايعات بدن در پرسنل با سابقه كاري 15-10 سال (3/73%) و بیش­ترین موارد تماس در مقایسه گروه­های شغلی مختلف با هم در پزشکان عمومی (100%)، ماماها (%4/82) و تکنسین­های هوشبری (75%) دیده شد. 8/83% افراد تحت مطالعه سابقه واكسيناسيون را ذكر نمودند كه از اين تعداد فقط 63 نفر(33%) تیتر آنتی­بادی خود را بررسي نموده بودند و تنها 9 نفر از تیتر خود اطلاع داشتند. 5/54% از افراد سابقه تماس با خون و مايعات بدن را ذکر نموده بودند که بیش­ترین موارد مربوط به فرو رفتن سوزن حین نمونه­گیری و دفع پسماند (5/46%) بود. میزان اطلاع­رساني و اقدامات بعدي بسيار ضعيف (12%) بود. 2/59% از افراد، استفاده از دستکش و 9/79% اطلاع از قوانین بهداشتی حین کار را ذکر نموده بودند. خوشبختانه تمامي افراد شركت­كننده در مطالعه از نظر  HCV-Ab و  HBS-Agمنفي بودند. سطح HBs-Ab در 63% موارد بالای 10 و در 31% از آن­ها بالای 100 واحد بين­المللي در ميلي ليتر بود. نتیجه­گیری: ميزان تماس پرسنل بيمارستان با خون و مايعات بدن بالا می­باشد. میزان عدم ايمن­سازي آن­ها در مواجهه با حوادث شغلی قابل توجه بوده و توجه به سلامت آن­ها همراه با اقدامات پيشگيرانه و پيگيري جدي در موارد حادثه­هاي شغلي و راه­اندازي مراكز سلامت شغلي از ملزومات هر بيمارستان مي­باشد.

Title: پیش¬گویی زخم فشاری در بیماران بستری با استفاده از معیارهای برادن و واترلو: یک مطالعه مقایسه ای

http://knh.shmu.ac.ir/index.php/site/article/view/161

مقدمه: هدف از مطالعه حاضرتعیین و مقایسه ابزارهای پیشگویی­کننده برادن و واترلو و هم­چنین تعیین نقطه برش این ابزارها در تشخيص زخم فشاري می­باشد. مواد و روش­ها: مطالعه حاضر یک مطالعه مقطعی است که بر روی 1864 بیمار بستری در بیمارستان امام حسين (ع) شاهرود انجام گردید. ابزار گردآوری شامل: ابزار ارزیابی پوست جهت تعیین زخم در بیماران و ابزارهای ارزیابی خطر برادن و واترلو می­باشد. اطلاعات از طریق مصاحبه و مشاهده جمع­آوری گردید و از طریق آزمون T و نمودار ROC مورد تجزيه و تحليل قرار گرفت. نتايج: از 1864 بیمار مورد بررسی 72 نفر به زخم فشاری مبتلا شدند. میانگین نمرات ارزیابی زخم در مبتلایان بر اساس معیار برادن 6/3±8/13 و در گروه غیر مبتلا 5/3±2/20 می­باشد و میانگین نمرات براساس معیار واترلو در مبتلایان 2/5±6/15 و در بیماران بدون زخم 1/4±10 می­باشد که اختلاف ميانگين نمرات دو گروه بيماران بر اساس هر دو معيار به طور معناداري متفاوت گزارش شده است (001/0P<). بر اساس نمودار راك، معیار برادن دارای حساسیت 92% و ویژگی 74% در نقطه تشخیصی 5/18 است و معیار واترلو در نقطه تشخیصی 5/12 به بالا دارای حساسیت 69% و ویژگی 78% می­باشد.نتیجه­گیری: معیار برادن در مقایسه با معیار واترلو از حساسیت و ویژگی بالاتری برخوردار است و یکی از ابزارهای مناسب برای تعیین بیماران در معرض خطر زخم فشاري می­باشد
